# Inflammation in Pulmonary Hypertension and Edema Induced by Hypobaric Hypoxia Exposure

**DOI:** 10.3390/ijms232012656

**Published:** 2022-10-21

**Authors:** Samia El Alam, Eduardo Pena, Diego Aguilera, Patricia Siques, Julio Brito

**Affiliations:** 1 Institute of Health Studies, Arturo Prat University, Iquique 1100000, Chile; 2 Institute DECIPHER, German-Chilean Institute for Research on Pulmonary Hypoxia and Its Health Sequelae, Hamburg (Germany) and Iquique (Chile), Avenida Arturo Prat 2120, Iquique 1110939, Chile

**Keywords:** inflammation, high altitude, pulmonary edema, pulmonary hypertension, hypoxia

## Abstract

Exposure to high altitudes generates a decrease in the partial pressure of oxygen, triggering a hypobaric hypoxic condition. This condition produces pathophysiologic alterations in an organism. In the lung, one of the principal responses to hypoxia is the development of hypoxic pulmonary vasoconstriction (HPV), which improves gas exchange. However, when HPV is exacerbated, it induces high-altitude pulmonary hypertension (HAPH). Another important illness in hypobaric hypoxia is high-altitude pulmonary edema (HAPE), which occurs under acute exposure. Several studies have shown that inflammatory processes are activated in high-altitude illnesses, highlighting the importance of the crosstalk between hypoxia and inflammation. The aim of this review is to determine the inflammatory pathways involved in hypobaric hypoxia, to investigate the key role of inflammation in lung pathologies, such as HAPH and HAPE, and to summarize different anti-inflammatory treatment approaches for these high-altitude illnesses. In conclusion, both HAPE and HAPH show an increase in inflammatory cell infiltration (macrophages and neutrophils), cytokine levels (IL-6, TNF-α and IL-1β), chemokine levels (MCP-1), and cell adhesion molecule levels (ICAM-1 and VCAM-1), and anti-inflammatory treatments (decreasing all inflammatory components mentioned above) seem to be promising mitigation strategies for treating lung pathologies associated with high-altitude exposure.

## 1. Introduction

Studies have shown a strong relationship between the inflammatory process and the progression or aggravation of different pathologies [1,2,3,4], and there is evidence for crosstalk between hypoxia and inflammation that shows positive feedback between the stabilization and expression of hypoxia-inducible factor-1α (HIF-1α) and several inflammatory molecules [5,6]. Specifically, in response to hypobaric hypoxia, which is generated by high altitude exposure (>2500 m) and the decrease in the partial pressure of oxygen, the inflammatory process is rapidly activated in human subjects and animal models [7,8].

One of the principal responses in the lung to hypobaric hypoxia conditions is hypoxic pulmonary vasoconstriction (HPV). The physiological effects of HPV include pulmonary artery vasoconstriction, which redistributes the blood stream toward more ventilated areas of the lung, improving ventilation/perfusion matching and oxygen uptake [9,10]. However, when HPV is permanent, it activates molecular pathways that trigger pulmonary artery remodeling and, consequently, high-altitude pulmonary hypertension (HAPH). HAPH is classically defined as an increase in the mean pulmonary artery pressure (mPAP) of 30 mmHg or more [11,12].

Another pathology related to exacerbated HPV and subsequent HAPH under hypobaric hypoxic conditions is high-altitude pulmonary edema (HAPE), which is a noncardiogenic, acute, and potentially lethal pulmonary alteration [13]. HAPE is characterized by an increase in pulmonary arterial pressure and vasoconstriction. These conditions increase vascular permeability in alveolar capillaries and ultimately aggravate hypoxic conditions [14,15,16].

The central effector mechanism of HPV is found within pulmonary artery smooth muscle cells (PASMCs) [10]. Recent work summarizes the alterations in molecular pathways in PASMCs that are related to HAPH, and inflammatory pathways seem to be important contributors to PASMC proliferation and pulmonary hypertension [17]. In addition to HAPH, HAPE may also be related to the inflammatory process; however, the latter exhibits some controversies, because some studies showed that inflammation could play a role in lung permeability in patients with HAPE [18], while others studies indicated that in humans, inflammation may be a secondary response to alveolar-capillary barrier disruption or edema [19].

Based on these findings, different studies have been performed to investigate preventative approaches for these pathologies related to high-altitude exposure, and the role of inflammation in response to these conditions was also examined [20,21,22]. Therefore, the aims of this review are to highlight the inflammatory pathways associated with hypobaric hypoxia, emphasize the key role of inflammation in lung pathologies, such as HAPH and HAPE, contribute to the understanding and identification of new biomarkers related to these high-altitude illnesses, and summarize different current anti-inflammatory approaches.

## 2. Hypoxia and Inflammation

In general, hypoxia can cause inflammation in an organism primarily by stimulating nuclear factor kappa B (NF-κB) gene transcription and the production of proinflammatory cytokines. Moreover, the reverse is also true since inflamed tissue can also become hypoxic [23]. The primary molecular responses to hypoxia are mediated by the transcription factor hypoxia-inducible factor (HIF). HIF regulates more than 200 genes and is present in several isoforms. Under hypobaric hypoxic conditions, the most studied isoform is HIF-1α [24,25].

Under normoxic conditions, HIF is hydroxylated at two proline residues through the prolyl hydroxylase (PHD) enzyme, where the Von Hippel–Lindau (VHL) ubiquitin ligase complex induces prolyl-hydroxylated HIF subunit degradation. However, under any hypoxic conditions, PHD hydroxylation activity is inhibited. This stabilizes HIF in the specific tissue or organism and contributes to the hypoxic mechanism [26]. Moreover, HIF-1α can also be stabilized by inflammatory molecules, such as IL-1β, NF-κB and TNF-α. NF-κB has been considered the master regulator of inflammation under hypoxic conditions since NF-κB can stabilize HIF-1α when it is released from inhibitory kinase b (IKb) through nuclear factor kinase subunit b (Ikkβ) activation [27]. Furthermore, a study showed that the regulatory protein of HIF, prolyl hydroxylase domain enzymes 2 (PHD2), serves as a coactivator of NF-κB in a HIF-1-independent fashion [28].

There is also evidence of crosstalk between HIF and the glucocorticoid receptor (GR). Glucocorticoids (GCs) are steroid hormones that play an important role in inflammation since they limit the production of most cytokines, such as IL-1, IL-2, IL-3, IL-4, IL-5, IL-6, IL-8, IL-12, IL-18, TNF-α, and interferon-γ (INF-γ), to maintain homeostasis [29,30]. A study showed the upregulation of GR in human proximal tubular epithelial cells might occur through the binding of HIF-1α to one or more hypoxia responsive element (HRE) sites in the nuclear receptor subfamily 3, group C, member 1 (*NR3C1)* promotor, and it thereby enhances GR transcription [31]. However, in human alveolar epithelial cells exposed to hypoxia, there was a downregulation of both the mRNA and protein levels of GR [32] and the inhibition of the nuclear translocation of GR [33]. Interestingly, a study conducted by Vettori et al. [34] showed that GC can stabilize HIF-1α through inactivation of the VHL complex.

**Figure 1:**
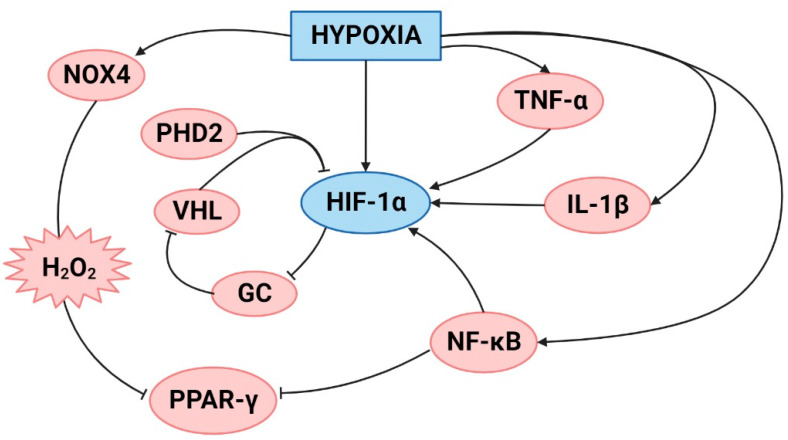
Proposed summary of the crosstalk between hypoxia and inflammation. Created with BioRender.com.

Moreover, hypoxia also increases the production of ROS and thus oxidative stress, and this condition is closely related to the inflammatory process [3]. For example, studies under normobaric hypoxic conditions showed that pulmonary artery hypertension is induced by an increase in NF-κB through the activation of ERK-1/2, which produces oxidative stress via Nox4-generated H_2_O_2_. In this process, ERK1/2, NF-κB, and H_2_O_2_ contribute to the inhibition of peroxisome proliferator-activated receptor y (PPARγ), which triggers pulmonary hypertension [35]. Under hypobaric hypoxic conditions, the crosstalk between oxidative stress and inflammation is important. Studies have demonstrated positive feedback between oxidative stress and inflammation, which recently has been coined “oxinflammation” [36,37]. Therefore, based on the mentioned evidence, there is an important role of inflammation under hypoxic conditions that could affect different tissues and organs, including the lung (Figure 1), which will be discussed below.

## 3. Lung Inflammation under Hypobaric Hypoxia

High-altitude or hypobaric hypoxia exposure can affect the immune homeostatic mechanism and immunoregulatory activities that may lead to several pathologies associated with this hypoxic condition [38]. Additionally, there is evidence that exposure to hypobaric hypoxia induces an inflammatory response [7]. Moreover, a study demonstrated an increase in the levels of IL-6 and the inflammatory biomarker IL-1 receptor antagonist in the serum of subjects exposed to high altitude, with a peak increase at Day 2 of exposure. Additionally, C-reactive protein (CRP) was increased in serum after 3 days of exposure [7]. In addition, a recent study in rats exposed to hypobaric hypoxic conditions for 72 h showed an increase in IL-1β in serum levels [21]. Therefore, it is important to highlight that there is an early inflammatory response after exposure to hypobaric hypoxia.

A moderate increase in circulating cytokine levels may reflect substantial inflammation in specific tissues, such as the lungs. Studies have demonstrated that under acute hypobaric hypoxia (3 h; 7620 m), there is an increase in the serum levels of proinflammatory cytokines (IL-1β, IL-6 and TNF-α) and neutrophil infiltration in the lungs of rats [39]. This is corroborated by a recent study that demonstrated an increase in inflammatory cell infiltration in the lungs of mice exposed to acute hypobaric hypoxia (7000 m; 7 days) [40]. Moreover, a recent study showed that the expression of inflammation-related genes (*MMP8*, *MMP9*, *IL-17β*, and *Timp1*) was upregulated in the lungs of rats exposed to the same conditions of hypoxia (acute hypobaric hypoxia) [21].

Additionally, the levels of inflammatory cytokines (TNF-α, IL-1β, and IL-6) were higher in the bronchoalveolar lavage of rats exposed to acute hypobaric hypoxia (6 h; 5000 m) plus inflammation induced by administration of lipopolysaccharides (LPS) than in that for each stimulus alone. Moreover, RT-qPCR data suggested that the combined stimulation of LPS and hypobaric hypoxia had a synergistic effect on gene expression levels in acute lung injury. This finding indicates that the promoter activity of toll-like receptor 4 (TLR4) was higher in the LPS plus hypobaric hypoxia group. Additionally, the inhibition of HIF-1α increased the promoter activities of the TLR4 gene, showing the importance of the crosstalk between the HIF-1α and TLR4 pathways. The synergistic effect of inflammation and hypoxia exposure may be critical in the development of HAPE. Therefore, people with upper respiratory tract infections should avoid high-altitude exposure [41].

Regarding chronic hypobaric hypoxia, a study in rats also determined an increase in TNF-α expression in the lung tissue [42]. Additionally, a study in mice showed an increase in macrophages in the lung over the course of hypobaric hypoxia exposure, with a peak at Day 21 [43]. In fact, based on epidemiological studies of hypobaric hypoxia, there is an increase in both the genetic and protein levels of inflammatory biomarkers, such as IL-6, TNF-α, IL-1β, and IL-1α, and these biomarkers are present in different populations exposed to high altitudes (Andean and Tibetans) [44,45].

Particularly, the expression of IL-8 under this condition is controversial. Studies involving humans exposed to hypobaric hypoxia showed no changes in IL-8 levels in serum [38], but a previous study in endothelial cell cultures demonstrated an increase in this cytokine after 16 hours of exposure to hypobaric hypoxia [46]. Additionally, a study using rats exposed to chronic hypobaric hypoxia showed that the level of IL-8 was increased in lung tissue from the first day of exposure. This expression decreased over time, but it was still higher than that in the control group [47]. Therefore, the expression of IL-8 might depend on the time of exposure and type of tissue analyzed.

**Figure 2:**
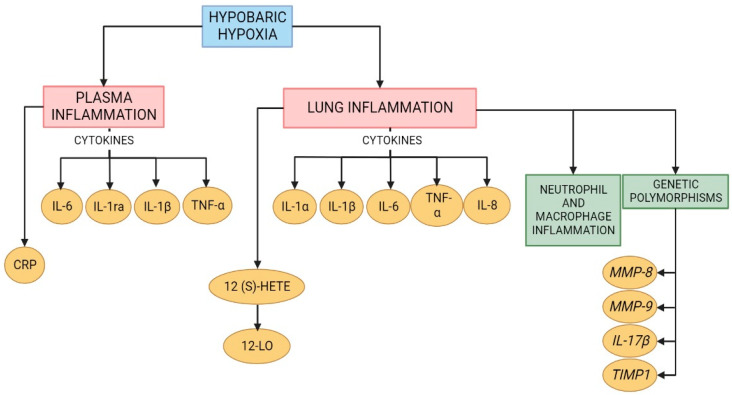
Schematic representation of the molecules involved in lung and plasma inflammation under hypobaric hypoxic conditions. Created with BioRender.com.

An interesting study in rats exposed to chronic hypobaric hypoxia showed an increase in 12(s)-hydroxyeicosatetraenoic acid (12(s)-HETE) expression in the lung, which was produced by leukocyte-type 12 lipoxygenase (12-LO) activation. This activation contributes to inflammatory pathways and the activation of ERK1/2 and p38 MAPK in smooth muscle cells (SMCs). Then, the proliferation process is stimulated, and HAPH subsequently develops [48]. Therefore, both acute and chronic exposure to high altitudes activate inflammatory pathways that contribute to the development of pulmonary high-altitude illnesses such as HAPE and HAPH (Figure 2), which will be discussed in the following sections.

## 4. High Altitude Pulmonary Edema

HAPE is a noncardiogenic pulmonary edema caused by pulmonary blood–gas barrier leakage in the lung. It can occur in rapidly ascending non-acclimatized individuals after arrival at altitudes above 2500 m, and profound hypoxemia and death may occur if the condition is not treated [49].

Inflammation might contribute to the pathogenesis of HAPE in susceptible individuals [7]. Although, Swenson et al. [50] claimed that HAPE is not related to inflammation since there were no significant differences in the bronchoalveolar lavage levels of leukocytes, cytokines (IL-1β, IL-8 and TNF-α), and eicosanoids between subjects resistant or susceptible to HAPE at high altitudes. However, subsequent studies have analyzed cytokines to assess the underlying mechanisms of the development of HAPE. Patients with HAPE have increased TNF-α and IL-6 levels in serum [51]. Then, Sharma et al. [18] determined that the levels of TNF-α were increased in the blood of individuals with HAPE, and this alteration could have a role in lung permeability in patients with HAPE.

Studies in animal models also showed that cytokine levels were increased by hypoxia. Rats exposed to acute hypobaric hypoxia with signs of HAPE showed a 13-fold increase in NF-κB levels (nuclear fraction), and NF-κB regulated the increase in inflammatory molecules (IL-1, IL-6, and TNF-α) in lung tissue under this hypoxic condition, highlighting an increase in the levels of the cell adhesion molecules ICAM-1 and VCAM-1 [13].

Additionally, another study in rats with HAPE due to acute hypobaric hypoxia (9142 m for 5 h) also showed that the levels of proinflammatory molecules, such as TNF-α, monocyte chemoattractant protein-1 (MCP-1), INF-γ, IL-6, and TNF-β, in the bronchoalveolar lavage were increased, and the levels of NF-κB in lung nuclear extracts were increased [52]. MCP-1 and macrophage inflammatory protein-1α (MIP-1α) are important immune response modulators that can be altered by high-altitude exposure [38].

A subsequent study demonstrated that the levels of soluble urokinase-type plasminogen activator receptor (suPAR), a biomarker of inflammation, were increased along with CRP and IL-6 levels in the plasma of subjects exposed to acute hypobaric hypoxia [53]. However, since not all cases of HAPE had evidence of inflammation in the alveolar lavage fluid, inflammation could be a secondary response to alveolar–capillary barrier disruption or edema [19]. This topic still needs further study.

The results of studies in HAPE-susceptible subjects showed an increase in proinflammatory chemokines (MIP-1α and MCP-1) compared with control subjects at sea level. In addition, an elevation of these molecules was demonstrated in HAPE-susceptible subjects before exposure to high altitudes (basal line). Moreover, the level of IL-8 was not significantly different in HAPE-susceptible subjects [38]. Additional studies have also demonstrated that suPAR plasma concentration levels were high in HAPE-susceptible subjects, suggesting that suPAR could serve as a possible biomarker of HAPE susceptibility. Moreover, the level of suPAR indicated that the subject susceptible to HAPE has a low-grade inflammatory condition; therefore, inflammation seems to modulate but not be the cause of HAPE [53].

At the genetic level, a study in Han Chinese subjects with and without HAPE reported 12 single nucleotide polymorphisms (SNPs) in the *NR3C1* gene between these groups; *NR3C1* encodes human GR. Moreover, it is important to note that these polymorphisms were significantly associated with the risk of HAPE [54].

**Figure 3:**
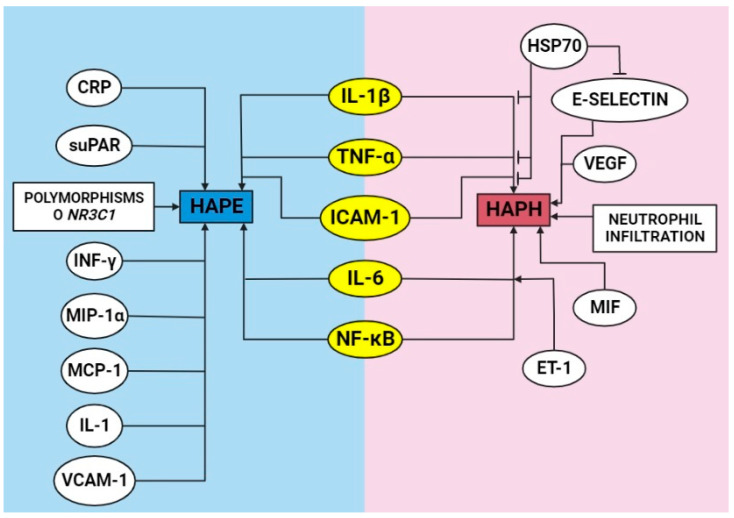
Schematic depiction of the proposed the inflammatory molecules and alterations involved in the development of high-altitude pulmonary edema (HAPE) and high-altitude pulmonary hypertension (HAPH). Created with BioRender.com.

The inflammatory basis of HAPE pathophysiology is still debatable; thus, studies with a larger number of subjects are recommended for better understanding [55]. Therefore, although the role of inflammation in HAPE is unclear, there is an important increase in proinflammatory cytokines and chemokines that might modulate the development of HAPE (Figure 3). Different treatment approaches have been studied to diminish these factors and will be discussed later.

## 5. High-Altitude Pulmonary Hypertension

As previously mentioned, one of the principal pathologies occurring under hypobaric hypoxia is HAPH. HAPH is a consequence of sustained hypoxic pulmonary vasoconstriction and the remodeling of pulmonary arteries mainly through proliferation of SMCs [12]. Pulmonary hypertension generates an increase in pressure load on the right ventricle, leading to right heart failure and eventually death [56].

Significant proliferation of vascular smooth muscle cells (VSMCs) has been observed within 24 h of hypoxic exposure [57]. This proliferation process could be conditioned by inflammation. A proteomic study in rats exposed to intermittent hypobaric hypoxia (5500 m) showed that heat shock protein 70 (HSP70) and protein disulfide isomerase associated 3 (PDIA3), regulators of inflammation, modulate the development of vascular remodeling that occurs due to HAPH [58]. Moreover, PDIA3 has been related to an increase in inflammatory molecules (IL-1β, IL-6, and TNF-α) in the brain [59] and lung [60].

Moreover, HSP70 presents anti-inflammatory properties and plays a protective role in lung injury and fibrosis by inhibiting proinflammatory cytokine expression [61]. Preconditioning with a low dose of hypobaric hypoxia increased HSP70 in rats, and this attenuated the increase in inflammatory biomarkers in serum (TNF-α, IL-1β, E-selectin, and ICAM-1) [62]. It is important to note that these results have also been described in HAPE under the same experimental conditions [63].

Another important proinflammatory cytokine is macrophage migration inhibitory factor (MIF), which was upregulated in the lungs of rats with chronic hypobaric hypoxia-induced pulmonary hypertension. Interestingly, MIF stimulates rat PASMC proliferation. This probably occurs through the ERK1/2 and JNK pathways, without the involvement of p38 MAPK [64]. Other studies in rats with pulmonary hypertension induced by similar hypobaric hypoxia conditions showed an important infiltration of neutrophils in the lung and robust expression of phosphorylated NF-κB, IL-6, IL-1β, TNF-α, and VEGF. All these factors were decreased after resveratrol treatment [65], and these treatments (Table 1) will be discussed in the next section.

Additionally, endothelin-1 (ET-1) is a relevant molecule that plays a crucial role in promoting hypoxia-induced pulmonary hypertension, and ET-1 is a vasoconstrictor peptide that is produced by endothelial cells [72,73]. A study in rats demonstrated that circulating and lung ET-1 levels increased after acute hypobaric hypoxia exposure and were related to hypoxic pulmonary vasoconstriction [66]. It is important to note that several studies have demonstrated that ET-1 is strongly related to lung inflammation and fibrosis [74,75,76]. In vitro experiments showed that stimulation with ET-1 significantly enhanced IL-6 expression in pulmonary arterial endothelial cells [73]. Additionally, peroxisome proliferator-activated receptor γ (PPARγ) inactivation showed increased ET-1-induced vascular injury, probably through pro-oxidant and proinflammatory pathways (Figure 3) [77].

## 6. Treatments

The etiology of pulmonary hypertension is an important factor to consider when developing a treatment study. Interesting research has demonstrated that the administration of cilostazol (a vasodilator inhibitor of phosphodiesterase-3) prevented the pulmonary hypertension induced by monocrotaline administration, but it was not effective in hypobaric hypoxia-induced pulmonary hypertension [78]. Additionally, it is important to note that the age of the experimental animal should be considered, as one study shows that the morphometric alterations in the lung induced by hypobaric hypoxia exposure are more exacerbated in older rats (24 months old) than in young rats (4 weeks old) [79].

Regarding anti-inflammatory approaches, Arya et al. [39] showed that inflammation signs in the lung of rats (increases in cytokines and neutrophil infiltration) were prevented by the administration of cerium oxide nanoparticles prior to hypobaric hypoxia exposure (7620 m; 6 h). In addition, pretreatment with netrine-1 (neuronal guidance protein-1) reduces neutrophil infiltration in the lungs of mice exposed to acute hypobaric hypoxia and attenuates the increase in macrophage inflammatory protein 2 (MIP-2) [69].

Dexamethasone is a synthetic glucocorticosteroid that is a potent anti-inflammatory drug that has also been used as a traditional prophylaxis for HAPE and other high-altitude illnesses, such as acute mountain sickness (AMS) and high-altitude cerebral edema (HACE) [19,80]. However, because of its diverse side effects [67,81], researchers have been proposing different anti-inflammatory approaches to reduce HAPE. A study determined that preconditioning with cobalt attenuated the pulmonary vascular leakage induced by hypobaric hypoxia in rats (9142 m; 5 h) and reduced inflammatory molecules, such as TNF-α/β, NF-κB, MCP-1, and IL-6 [52]. Another rat study showed that the administration of curcumin before hypobaric hypoxic exposure reduced signs of pulmonary edema and inhibited hypoxia-induced elevations in NF-κB levels [13]. A subsequent study in rats demonstrated that treatment with nanocurcumin was more effective than curcumin in reducing HAPE and pulmonary vascular medial wall thickness, and these changes were related to decreases in the levels of TNF-α, TNF-β, IL-6, and ET-1 in the plasma [66].

Other phytocompounds have been evaluated for the ability to reduce HAPE. Quercetin is a potent antioxidant and anti-inflammatory phytoflavonol. Administration of quercetin to rats 1 h prior to hypobaric hypoxia (7620 m; 6 h) exposure attenuated transvascular leakage after hypoxia exposure, downregulated NF-κB and TNF-α expression, decreased the expression of cell adhesion molecules (ICAM-1, VCAM-1, and P-selectin), and increased the expression of anti-inflammatory cytokines (TGF-β and IL-4) [67]. Pretreatment with *Potentilla anserina* L. polysaccharide in rats exposed to hypobaric hypoxia (8000 m; 72 h) ameliorated HAPE and decreased oxidative stress and the levels of proinflammatory cytokines (IL-1β, TNF-α, IL-6). In addition, hypobaric hypoxia increased the levels of HIF-1α and NF-κB, whereas pretreatment with this polysaccharide inhibited both transcription factors [68].

Regarding pulmonary artery hypertension induced by hypobaric hypoxic conditions, a study in rats under chronic hypobaric hypoxia determined that the oral administration of tadalafil, a phosphodiesterase-5 inhibitor considered a potent antioxidant, reduced oxidative stress, inflammation, and vasoconstriction. These three effects suggest that tadalafil could be considered a promising treatment option for pulmonary hypertension induced by hypobaric hypoxia [42]. A recent study in rats demonstrated that the administration of magnesium lithospermate B derived from *Salvia miltiorrhiza* reduces high altitude-induced pulmonary hypertension through the downregulation of HIF-1α, MCP-1, and NF-κB [70].

Furthermore, hypobaric hypoxia exposure could also be used as a treatment to minimize pulmonary pathologies. In a recent study, rats with monocrotaline-induced pulmonary artery hypertension were treated with chronic intermittent hypobaric hypoxia (6 h/day) for 4 weeks. In these rats, chronic intermittent hypobaric hypoxia attenuated PAH and remodeling, which was probably achieved by inhibiting the NF-κB/p38 MAPK pathway in the lung. In addition, the treatment reduced macrophage infiltration in lung tissue and proinflammatory cytokine expression (TNF-α and IL-6) [71]. A different study in rats showed that preconditioning with low doses of hypobaric hypoxia (1000 m, 5 h/day for 5 consecutive days for 2 weeks) reduces the pulmonary pathologies induced by hypobaric hypoxia exposure (6000 m; 24 h), such as HAPE, through a decrease in inflammatory molecules [63]. Therefore, we can hypothesize that preconditioning with low doses of hypobaric hypoxia could be a good strategy to abolish high-altitude pulmonary pathologies, which seems interesting and warrants further evaluation. Finally, it is important to note that anti-inflammatory studies of human subjects exposed to hypobaric hypoxia are rather scarce, and it is necessary to expand knowledge on this matter to improve treatment recommendations.

## 7. Conclusions

HAPE and HAPH induced by hypobaric hypoxia exposure are strongly related to increases in lung inflammation due to increases in inflammatory cell infiltration (macrophages and neutrophils), cytokine levels (IL-6, TNF-α and IL-1β), chemokine levels (MCP-1), and cell adhesion molecule levels (ICAM-1 and VCAM-1). These effects are reflected in systemic inflammation. The role of inflammation in the development of these diseases, specifically in HAPE, is not very clear in human studies, which could be considered a limitation and a new avenue for future research. Anti-inflammatory approaches, such as decreasing the previously mentioned inflammatory components, may be promising mitigating strategies for lung pathologies associated with high-altitude exposure and have been well evidenced in animal models, but more human studies or clinical trials are necessary to improve the prevention and treatment recommendations for these high-altitude illnesses. Therefore, this review suggests inflammatory target molecules that are involved in HAPE and HAPH pathologies, which could be considered as possible biomarkers in future studies. However, additional studies are needed to improve the understanding of the role of inflammation in high-altitude illnesses.

## Figures and Tables

**Table 1:** Anti-inflammatory treatments for lung alterations due to hypobaric hypoxia.

Treatment	Lung Injury	Animal Model	Anti-Inflammatory Effects	References
Cobalt	HAPE	Sprague Dawley rats	Decrease TNF-α, TNF-β, NF-κB, MCP-1, and IL-6/Increase HO-1 and MT	Shukla et al. [52]
Curcumin	HAPE	Sprague Dawley rats	Decrease NF-κB	Sarada et al. [13]
Nanocurcumin	HAPE	Sprague Dawley rats	Decrease TNF-α, TNF-β, IL-6, and ET-1	Nehra et al. [66]
Quercetin	HAPE	Sprague Dawley rats	Downregulate NF-κB and TNF-α Decreased ICAM-1, VCAM-1, and P-selectin Increase TGF-β and IL-4	Tripathi et al. [67]
* Potentilla anserina * L polysaccharide	HAPE	Wistar rats	Decrease IL-1β, TNF-α, IL-6 Inhibition HIF-1α and NF-κB	Shi et al. [68]
Hypobaric hypoxia preconditioning	HAPE	Sprague Dawley rats	Increase HSP70	Lin et al. [63]
Netrine-1	HAPI	Mice	Reduced neutrophil infiltration Decrease MIP-2	Ko et al. [69]
Cerium oxide nanoparticles	HAPI	Sprague Dawley rats	Decrease IL-1β, IL-6, and TNF-α	Arya et al. [39]
Tadalafil	HAPH	Wistar rats	Decrease TNF-α Decrease inflammatory cells infiltration	Rashid et al. [42]
Magnesium lithospermate B	HAPH	Sprague Dawley rats	Downregulated HIF-1α MCP-1 and NF-κB	Wang et al. [70]
Intermittent Hypobaric hypoxia treatment	HAPH	Sprague Dawley rats	Decrease NF-κB, TNF-α, and IL-6 and macrophage infiltration	Gao et al. [71]
Resveratrol	HAPH	Sprague Dawley rats	Decrease IL-6, IL-1β, TNF-α, VEGF, and HIF-1α	Xu et al. [65]

TNF-α, Tumor Necrosis Factor Alpha; TNF-β, Tumor Necrosis Factor Beta; HIF-1α, Hypoxia Inducible Factor-1α; MCP-1, Monocyte Chemoattractant Protein-1; NF-κB, Nuclear Factor-kappa B; IL-6, Interleukin-6; MIP-2, Macrophage Inflammatory Protein-2; IL-1β, Interleukin-1 beta; HO-1, Heme Oxygenase-1; MT, Metallothionein; ET-1, Endothelin-1; ICAM-1, Intercellular Adhesion Molecule-1; VCAM-1, Vascular Cell Adhesion Molecule-1; TGF-β, Transforming Growth Factor-beta; IL-4, interleukin-4; HSP70, Heat Shock Protein 70; HAPH, High Altitude Pulmonary Hypertension; HAPE, High Altitude Pulmonary Edema; HAPI, High Altitude Pulmonary Inflammation.

## Abbreviations

HIF-1α	hypoxia-inducible factor-1α
GR	glucocorticoid receptor
*MMP8*	matrix metalloproteinase 8
*MMP9*	matrix metalloproteinase 9
*IL-17β*	interleukin-17β
*TIMP1*	tissue inhibitor of metalloproteinase 1
*NR3C1*	nuclear receptor subfamily 3, group C, member 1
ICAM-1	intercellular adhesion molecule-1
VCAM-1	vascular cell adhesion molecule 1
NF-κB	nuclear factor kappa B
TNF-α	tumor necrosis factor alpha
TLR4	toll-like receptor 4
12(s)-HETE	12(s)-hydroxyeicosatetraenoic acid
12-LO	leukocyte-type 12 lipoxygenase
suPAR	soluble urokinase-type plasminogen activator receptor
MCP-1	monocyte chemoattractant protein-1
MIP-1α	macrophage inflammatory protein-1α
HSP70	heat shock protein 70
PDIA3	protein disulfide isomerase associated 3
MIF	macrophage migration inhibitory factor
ET-1	endothelin 1
PPARγ	proliferator-activated receptor γ
MIP-2	macrophage inflammatory protein 2
